# Patient Attitudes and Beliefs and Provider Practices Regarding Antibiotic Use for Acute Respiratory Tract Infections in Minya, Egypt

**DOI:** 10.3390/antibiotics3040632

**Published:** 2014-11-14

**Authors:** Amr Kandeel, Waleed El-Shoubary, Lauri A. Hicks, Mohamed Abdel Fattah, Kathleen L. Dooling, Anna Leena Lohiniva, Omnia Ragab, Ramy Galal, Maha Talaat

**Affiliations:** 1 Ministry of Health and Population, Cairo 11516, Egypt; E-Mails: kandeelamr@yahoo.com (A.K.); mohpaf@yahoo.com (M.A.F.); dr_omnya_ragab@yahoo.com (O.R.); Ramygalal_81@yahoo.com (R.G.); 2 U.S. Naval Medical Research Unit, No.3, Cairo 11517, Egypt; E-Mails: waleed.el-shoubary.eg@med.navy.mil (W.E.-S.); anna.leena.lohiniva@hotmail.com (A.L.L.); 3 Centers for Disease Control and Prevention, Atlanta, GA 30333, USA; E-Mails: auq3@cdc.gov (L.A.H.); kathleen.dooling@peelregion.ca (K.L.D.)

**Keywords:** antibiotics, acute respiratory infection, cold, bronchitis, sinusitis, pneumonia, developing countries, Egypt

## Abstract

The inappropriate use of antibiotics in the community is one of the major causes of antimicrobial resistance. This study aimed to explore the physician prescribing pattern of antibiotics for acute respiratory infections (ARIs) and to explore the knowledge, attitudes, and practices of patients regarding antibiotic use for ARIs. The study was conducted in Upper Egypt and used quantitative and qualitative research techniques. Eligible patients exiting outpatient clinics with ARIs were invited to participate in the study. A qualitative study was conducted through 20 focus group discussions. Out of 350 encounters for patients with various ARIs, 292 (83%) had been prescribed at least one antibiotic. Factors significantly associated with antibiotic prescribing for adults included patient preference that an antibiotic be prescribed. For children younger than 18, presentation with fever, cough, loss of appetite, and sore throat, along with the caregiver’s antibiotic preference, were associated with an antibiotic prescription. Several misconceptions regarding antibiotic use among community members were stated, such as the strong belief of the curing and prophylactic power of antibiotics for the common cold. Interventions to promote proper antibiotic use for ARIs need to be piloted, targeting both physicians and the public. Educational programs for physicians and campaigns to raise public awareness regarding proper antibiotic use for ARIs need to be developed.

## 1. Introduction

The inappropriate use of antibiotics is a worldwide public health problem contributing to the emergence of antimicrobial-resistant pathogens [1]. The resulting antimicrobial resistance is a major threat to public health, particularly in low and middle income countries, where the leading causes of illness and death are infectious [2]. In addition, access to antibiotics is generally unregulated in these countries, and some studies have shown that their misuse is widespread [3,4,5], especially unnecessary antibiotic treatment for respiratory illnesses [6,7,8]. In the early 1990s in the U.S., a high proportion of antibiotic prescriptions for both children and adults were for upper respiratory tract infections and bronchitis, for which these drugs provided no benefit [9,10]. Since then, much research has been conducted on trends and determinants of antibiotic prescribing in the U.S. [10,11,12,13,14], and the results have led to interventions aimed at improving antibiotic prescribing practices [15,16]. However, little is known about the frequency of antimicrobial use and misuse for upper respiratory infections and the factors that drive antibiotic use in Egypt.

The objectives of the study were to examine physician antibiotic prescribing practices for acute respiratory tract infections (ARIs) in outpatient clinics of hospitals and primary care units. We also aimed to explore the knowledge, attitudes, and practices of patients regarding antibiotic use for ARIs, and to identify cultural and societal determinants contributing to the use of antibiotics in Egypt.

## 2. Methods

### 2.1. Study Location

The study was conducted from May–July 2011 in Minya district, Upper Egypt. Minya district (population 220,000) is the capital of Minya Governorate, located 280 km south of Cairo. The majority of the population lives in rural areas where agriculture is the predominant economic activity. Minya district has a network of government health care facilities composed of tertiary-care hospitals, district hospitals, rural hospitals, and primary health care units. Minya was chosen by the Ministry of Health and Population to conduct baseline studies exploring the extent of antibiotic use in Egypt and evaluate the impact of a pilot intervention to reduce unnecessary prescription and consumption of antibiotics.

### 2.2. Quantitative Study

A survey was conducted at acute care hospitals and primary healthcare centers. Patients exiting outpatient clinics for internal medicine, pediatrics, and chest medicine; Ear, Nose, and Throat clinics; or general outpatient clinics in primary healthcare centers were screened through eligibility criteria for inclusion in the study. Screening was done by reviewing each patient’s prescription; patients with a physician diagnosis of common cold, otitis media, sinusitis, bronchitis, pharyngitis, influenza, or pneumonia were invited to be interviewed after consent. Adults were interviewed; children less than 18 years of age were represented by a parent or caretaker. Eight trained field investigators conducted a full-day visit to each of the selected healthcare facilities to interview all eligible patients during the day. A standardized data collection form was used for interviewing. Information collected included demographic characteristics (age, gender, and educational level), presenting symptoms, and characteristics of the current illness. Type and dosage of antibiotics prescribed were recorded on the questionnaire from the patient encounter. Additional information collected included patient knowledge, attitudes, and beliefs regarding antibiotic use.

A sample size was estimated to allow for a subsequent study evaluating the impact of an educational intervention to reduce antibiotic use. The sample size was determined based on the predicted change in the prescribing behaviors of physicians as a result of the intervention. Assuming a change in prescribing behavior of 20%, a physician prescribing rate of antibiotics of 60% for acute respiratory infections, a significance level of 95% (alpha 0.05) and a power (Beta) of 90%, a total of 231 patients was required. A comprehensive list of governmental healthcare facilities that provide care for outpatients with respiratory diseases was provided by Minya Health Directorate. All four available acute care hospitals in Minya district were visited for one full working day. In addition, out of 42 primary healthcare units available in Minya, 50% were selected randomly and visited for patient exit interviews.

Data were analyzed using the Statistical Package for the Social Sciences (SPSS) [17]. Descriptive statistics were used to summarize patient demographic characteristics, frequency of antibiotic prescription or use, and patient attitudes and beliefs regarding antibiotic usage. The influence of demographic characteristics and other factors on antibiotic usage was tested using Chi Square or Fisher’s exact tests when appropriate. The level of statistical significance was set at *p* < 0.05.

### 2.3. Qualitative Study

The qualitative study was undertaken in the village of Nazlet El Fellaheen (Minya district, population 9065). A total of 20 focus group discussions (FGDs) including 160 participants were conducted, equally divided among groups of women or men with higher or lower education levels. A less educated respondent was defined as a person with fewer than six years of schooling. A more educated respondent was defined as a person having either a diploma or university degree (12–16 years of education). Field workers experienced in qualitative research tools facilitated the focus group discussions after extensive training on the study objectives. The interviews were tape recorded, transcribed, and translated from Arabic to English. A qualitative research consultant did the data analysis of the transcribed text based on thematic analysis, which involved searching for meaningful segments of text and organizing them into categories based on emerging themes related to antibiotic use. The coded data was retrieved from the transcripts and placed in charts with quotes that allowed comparison across respondents within the target groups and across the groups. In the final stage, the data set was interpreted as a whole to answer the research questions [18].

## 3. Results and Discussion

### 3.1. Results

#### 3.1.1. Results of Quantitative Study

##### 3.1.1.1. Demographic Patient Characteristics

During the study period, a total of 387 patients were eligible for inclusion in the study; 350 (90%) consented to participate. A total of 64.8% (227/350) of the respondents were caregivers of children under 18. For adult patients, the median age was 37 years (range, 18–78 years), and about 60% were female. Half of the adults (47%) did not receive any formal education. Thirty percent of adults had history of chronic conditions, mostly diabetes mellitus, hypertension, and liver and kidney diseases. The median age for children under 18 was 3.5 years (range, 2.5 months–18 years), and 12.8% had chronic diseases (e.g., bronchial asthma, sickle cell anemia, and kidney or liver diseases). The description of the cohort of children under 18 and adults is presented in Table 1.

**Table 1:** Demographic characteristics of patients interviewed following a physician consultation.

Characteristic	Children under 18 years (n = 227)		Adult Patients (n = 123)	
	No.	%	No.	%
Age (years)				
Mean age ± SD	4.8 ± 4.1		40 ± 15	
Median (Range)	3.5 years (2.5 months–18 years)		37 years (18–78 years)	
Age groups				
≤5 y	150	66.1	---	---
6–20 y	77	33.9	20	16.4
21–40 y	---	---	53	43.4
41–60 y	---	---	37	30.3
≥60 y	---	---	12	9.8
Gender				
Male	113	49.8	45	36.6
Female	114	50.2	78	63.4
Educational Level				
No formal education	*		58	47.2
Elementary/primary school	*		23	18.7
Secondary school	*		32	26.0
University (bachelor’s degree)	*		10	8.1
Chronic Disease				
Yes	29	12.8	37	30.1
No	198	87.2	86	69.9

* Education level of children not collected.

**Table 2:** Antibiotic prescribing frequency for acute respiratory tract infections according to clinical diagnosis, Minya district, Egypt, 2011.

	Child Patients (under 18 years) (*n* = 227)		Adult Patients (*n* = 123)		
	# of Encounters	# and Percent of Encounters Resulting in an Antibiotic Prescription No. (%)	# of Encounters	# and Percent of Encounters Resulting in an Antibiotic Prescription No. (%)	
Ear Infections	2	2 (100)	4	4 (100)	
Tonsillitis	35	34 (97.1)	10	10 (100)	
Pharyngitis	53	51 (96.2)	27	26 (96.3)	
Sinusitis	7	6 (85.7)	10	8 (80)	
Bronchitis	78	63 (80.8)	35	33 (94.3)	
Common Cold	43	23 (53.5)	27	17 (63)	
Pneumonia	9	8 (88.9)	10	7 (70)	
Total	227	187 (82.4)	123	105 (85.4)	

**Table 3:** Univariate factors associated with antibiotic prescribing for ARIs (excluding pneumonia), Minya District, Egypt, 2011.

Characteristic	Child Patients (under 18 years) Prescribed Antibiotics No. (%)	χ2 *p*	Adult Patients Prescribed Antibiotics No. (%)	χ2 *p*
Healthcare facility type				
Acute care hospitals	109 (90.8)	<0.01	53 (88.3)	>0.05
Primary healthcare units	70 (72.2)		45 (84.9)	
Age groups				
≤5 y	114 (79.2)	>0.05	--	
6–20 y	65 (89)		15 (78.9)	>0.05
21–40 y	--		40 (81.6)	
41–60 y	--		30 (93.8)	
≥60 y	--		12 (100)	
Gender				
Male	87 (80.6)	>0.05	36 (92.3)	>0.05
Female	92 (84.4)		62 (83.8)	
Educational level				
No formal education	75 (83.3)	>0.05	48 (92.3)	>0.05
Elementary/primary school	36 (76.6)		18 (81.8)	
Secondary school	62 (84.9)		24 (82.8)	
University	6 (85.7)		8 (80)	
Presenting symptoms				
Fever				
Yes	118 (88.7)	<0.01	22 (88)	>0.05
No	61 (72.6)		76 (86.4)	
Cough				
Yes	119 (77.3)	<0.05	58 (89.2)	>0.05
No	60 (95.2)		40 (83.3)	
Loss of appetite				
Yes	114 (88.4)	<0.01	---	---
No	65 (73.9)		---	---
Sore throat				
Yes	106 (93)	<0.01	58 (85.3)	>0.05
No	54 (69.2)		40 (88.9)	
Difficulty in breathing				
Yes	50 (82)	>0.05	---	---
No	116 (84.7)		---	---
Runny nose				
Yes	102 (81.6)	>0.05	37 (86)	>0.05
No	77 (83.7)		61 (87)	
Caregiver or patient preference to be treated by an antibiotic				
Yes	109 (88.6)	<0.01	59 (95.2)	<0.05
No	34 (68)		23 (79.3)	

##### 3.1.1.2. Antibiotic Prescribing

Out of 350 encounters for patients with ARIs, 292 (83.4%) received at least one antibiotic (82% of children and 85% of adults). The antibiotic prescription rates for the various diagnostic categories for children and adults are described in Table 2. Penicillins were the most commonly prescribed antibiotics for ARIs among adults (33% of prescriptions), followed by cephalosporins (23.9%) and tetracyclines (23.9%). A high percentage (60%) of adult patients diagnosed with bronchitis was treated with tetracyclines, whereas 46% of adult patients with pharyngitis were treated with cephalosporins. Patients under 18 were most often treated with penicillins for bronchitis (47.6%), sinusitis and/or otitis media (62.5%), pharyngitis (41.2%), and the common cold and influenza (39%).

##### 3.1.1.3. Factors Associated with Prescribing Antibiotics

Several factors were significantly associated with high physician antibiotic prescribing rates on bivariate analysis for patients under 18 as well as adult patients (Table 3). For children attending clinics of acute care hospitals, presentation with fever, cough, loss of appetite, sore throat, or a caregiver preference that an antibiotic be prescribed, were associated with an antibiotic prescription for acute respiratory infection. For adult patients, the only factor significantly associated with receipt of an antibiotic prescription was the patient preference that an antibiotic be prescribed.

##### 3.1.1.4. Beliefs of Patients regarding Antibiotic Use

A majority of respondents (54% of caregivers and 62% of adult patients) believed that overuse of antibiotics is a major problem. More than 50% of survey respondents also believed that too many people are treated with antibiotics when not necessary, and almost 90% of both groups (caregivers and adult patients) believed that physicians should not prescribe antibiotics when not indicated. About 50% of the cohort agreed that treatment with antibiotics is necessary when nasal discharge turns from yellow to green. Many caregivers (45%) and adult patients (35%) believed that antibiotics can be used to prevent the common cold. A total of 61% of caregivers and 44% of adult patients believed that antibiotics help cold symptoms clear up quickly.

#### 3.1.2. Results of Qualitative Study

The practice of persons treating themselves with antibiotics for ARIs was very common and was mentioned by almost all participants of the focus groups. They had purchased antibiotics based either on the advice of pharmacists or that of neighbors or family. The results of the study identified four prevailing cultural beliefs influencing the use of antibiotics. These included beliefs regarding the effectiveness of antibiotics, perceived factors associated with effectiveness, the use of antibiotics as preventive medicine, and fear regarding overuse of antibiotics. Themes regarding beliefs and perceptions regarding antibiotic use did not appear to differ between higher and lower educated groups or between genders.

##### 3.1.2.1. Effectiveness of Antibiotics

Respondents expressed a strong belief in the curing power of antibiotics, describing antibiotics as “*powerful and magic medicines*” that lead to rapid cure. They believed that antibiotics were the only tools to cure most infectious diseases and acute illnesses. Some respondents also believed that treatment with antibiotics leads to better health of the patient.

“Nothing works like antibiotics. They have a great effect on any disease or illness.”

##### 3.1.2.2. Perceived Factors Associated with the Effectiveness of Antibiotics

Respondents believed that the dose, route of administration and cost of the antibiotic were associated with the effectiveness and rapidity of cure. Injectable antibiotics were preferred over oral antibiotics, because they were considered to act more rapidly and effectively to cure infection as they flow directly in the bloodstream. Respondents favored more costly antibiotics, stating that a higher price indicated good quality medicines that should be used for severe illnesses or after surgery. “*Cheap antibiotics have little effect on cure.*”

##### 3.1.2.3. Antibiotics as Preventive Medicine 

Many respondents believed that antibiotics can prevent illnesses, and therefore they reported initiating antibiotic treatment early after the onset of minor symptoms such as cough, especially among children. Respondents mentioned that antibiotics are usually kept at home to be used immediately for any sudden onset of symptoms of respiratory infection.

“*We don’t wait; we don’t risk getting sicker; we take antibiotics whenever we have any illness symptoms so microbes stay away.*”

“*If I feel tired after returning from work in the field, I may just take an antibiotic to make sure that I won’t get sick and will be able to work the next day.*”

##### 3.1.2.4. Fear of Overuse of Antibiotics

Respondents believed that the overuse of antibiotics could negatively impact their health by reducing their immune status or making them resistant to the antibiotic treatment for future infections. This argument indicated a preference for use of short courses of antibiotics, or stopping the antibiotic once symptoms started to resolve. Respondents also believed that using prophylactic single shots of antibiotics on a weekly or monthly basis was a better option compared to a long course of antibiotics, often prescribed by physicians for treatment of respiratory infections.

“*There is a possibility that antibiotics don’t work if we use them too much. I prefer taking one injection of antibiotics whenever I need it. If I feel better, I will not go for a second one. It is better to use them moderately.*”

### 3.2. Discussion

This is one of the first studies from a developing country in the Eastern Mediterranean Region (EMR) describing the prescribing practices of physicians and the perception of community members regarding antibiotic use for ARIs. The study findings revealed that physician prescriptions of antibiotics for ARIs were extremely high in Minya, Egypt, ranging from 53% for the common cold to greater than 95% for pharyngitis, tonsillitis, and ear infections. The vast majority of these infections are caused by viruses and do not require antibiotic treatment [19]. These figures are consistent with another study conducted in the same district (Minya), where 64% of physicians reported prescribing antibiotics either most of the time or sometimes for treatment of the common cold [20].

The data in this report confirm the findings of other studies demonstrating that antibiotic prescribing for the common cold is high. The high prescription rates of antibiotics for the common cold (53% for children under 18 and 63% for adults) were comparable to those from a study from Jordan, where more than 51% of patients with the common cold received a physician’s prescription including antibiotics [21]. As limited studies are available in the region, we compared our findings with studies in the U.S. in the late 1990s, before the launch of a national campaign to improve antibiotic prescribing, in which physician prescription of antibiotics was as high as 60% for patients with upper respiratory infections or the common cold [9,22,23]. The prescription rate for acute bronchitis in our study for adult patients (94%) and for children (80%) was higher than that in previous studies conducted in primary health care settings in the U.S. and the U.K. in the late 1990s, where it was reported that up to 75% of patients with acute bronchitis received antibiotics [9,16,24,25].

The tendency to prescribe antibiotics for the common cold in Egypt may have similar drivers that lead to overprescribing in high-income countries, including patient demand, diagnostic uncertainty, and limited time [26]. Physicians in acute care hospitals have been found to significantly prescribe at higher rates than primary care physicians. Previous studies have found that various physician specialties prescribe differently [24]. Physician prescription of an antibiotic was significantly associated with the caregiver or patient’s preference for receiving an antibiotic. Several studies have shown that the patient’s preference is an important determinant in antibiotic prescription and that antibiotics are more likely to be prescribed, even when they are not indicated, in order to satisfy the patient [21,25,27].

In a study conducted in the same district in Egypt, younger physicians were more likely to prescribe antibiotics for the common cold, in order to satisfy their patients and build a good reputation [20].

Antibiotic treatment of pneumonia was lower than expected (89% for children and 70% for adult patients). This is likely explained by the study sites and population; government facility pharmacies provide inexpensive, older antibiotics which physicians perceive to be less effective than newer antibiotics. It is likely that patients were advised to purchase newer antibiotics from private pharmacies to treat pneumonia. This finding should be explored further in future studies to better understand if there is under-treatment of pneumonia.

Almost all community members who participated in the FGDs harbored many misconceptions about antibiotic use that could contribute to inappropriate use. One of the most important findings was the belief that antibiotics are useful for prevention of the common cold, prompting early use of antibiotics for short durations to avoid developing a more severe infection. The perception that excessive antibiotic use leads to resistant organisms was not mentioned by the patients; rather, they thought that they themselves would develop resistance. Self-medication with antibiotics is facilitated by the availability of over-the-counter antibiotics in private pharmacies [20]. Similar misconceptions were noted in a study conducted in Jordan, where 67% of adults surveyed believed that antibiotics treat common colds and 55% used the antibiotics as prophylactic drugs against infections [21].

This study has several limitations. First, it was conducted in one district in Minya Governorate; thus the results may not be generalizable to areas outside Minya. Second, the diagnosis of ARIs was based on the physician’s judgment documented in the encounter, which may not be accurate; alternatively, the physician may have provided a diagnosis to justify antibiotic prescribing. Third, the presence of data collectors in the halls of the outpatient clinics might have influenced the prescribing practices of physicians, even though they did not know why the study was being conducted.

This study provides evidence that antibiotics are commonly overprescribed and consumed for treatment and prevention of ARIs in the Minya district, driven by both inappropriate prescribing by physicians and excessive demand by the public. Educational interventions targeting providers and the public in Minya district should be piloted to measure the impact of interventions on promoting proper antibiotic use for ARIs. More widespread studies on antimicrobial use at the community level need to be conducted to determine the extent of the problem in Egypt. Reduction of antibiotic use for ARIs should be a priority for the MOHP in Egypt, as evidence exists that the incidence of antimicrobial-resistant infections is positively correlated with outpatient antibiotic use [28]. Clinical treatment guidelines and prescribing policies for health care providers, particularly focusing on treatment of ARIs, as well as increased access to diagnostic testing (e.g., rapid streptococcal test), may improve appropriate antibiotic prescribing.

## 4. Conclusions

High inappropriate antibiotic prescription for ARIs existed in Minya. Several misconceptions regarding antibiotic use among community members were also stated. Data of this study could be used to design and pilot interventions to promote proper antibiotic use for ARIs targeting both physicians and the public.

## Disclaimer

Maha Talaat and Anna Leena Lohiniva are contractors of the U.S. Government. Waleed El-Shoubary is an employee of the U.S. Government. This work was prepared as part of their official duties. Title 17 USC × 105 provides that “copyright protection under this title is not available for any work of the United States Government.” Title 17 USC × 101 defines U.S. Government work as work prepared by a military service member or employee of the U.S. Government as part of that person’s official duties.
